# Facial threat affects trust more strongly than facial attractiveness in women than it does in men

**DOI:** 10.1038/s41598-021-01775-5

**Published:** 2021-11-18

**Authors:** Johanna Brustkern, Markus Heinrichs, Mirella Walker, Bastian Schiller

**Affiliations:** 1 Laboratory for Biological and Personality Psychology, Department of Psychology, University of Freiburg, Stefan-Meier-Str. 8, 79104 Freiburg, Germany; 2 Faculty of Psychology, University of Basel, Missionsstrasse 60/62, 4055 Basel, Switzerland

**Keywords:** Human behaviour, Psychology

## Abstract

Trust is essential in initiating social relationships. Due to the differential evolution of sex hormones as well as the fitness burdens of producing offspring, evaluations of a potential mating partner’s trustworthiness likely differ across sexes. Here, we explore unknown sex-specific effects of facial attractiveness and threat on trusting other-sex individuals. Ninety-three participants (singles; 46 women) attracted by the other sex performed an incentivized trust game. They had to decide whether to trust individuals of the other sex represented by a priori-created face stimuli gradually varying in the intensities of both attractiveness and threat. Male and female participants trusted attractive and unthreatening-looking individuals more often. However, whereas male participants’ trust behavior was affected equally by attractiveness and threat, female participants’ trust behavior was more strongly affected by threat than by attractiveness. This indicates that a partner’s high facial attractiveness might compensate for high facial threat in male but not female participants. Our findings suggest that men and women prioritize attractiveness and threat differentially, with women paying relatively more attention to threat cues inversely signaling parental investment than to attractiveness cues signaling reproductive fitness. This difference might be attributable to an evolutionary, biologically sex-specific decision regarding parental investment and reproduction behavior.

## Introduction

Trusting a stranger is a prerequisite for initiating any kinds of social relationships, especially those with potential mating partners^1–3^. Besides its obvious benefits for our personal well-being^4^, this particularly intimate sort of human social bond involves significant risks and interpersonal dependency, as the partner might choose to disappoint expectations or abuse one’s own investments in the relationship (e.g., emotions, time, resources^5,6^). To minimize such risks, it is necessary that we accurately and quickly infer another’s trustworthiness. In doing so, humans have evolved brain modules specialized in processing facial features, which are one of the richest sources of information in signaling social intentions^7,8^. Two facial features crucially affecting the decision whether or not to trust other-sex individuals are attractiveness and threat^9–13^. As sexual-selection forces operate differentially across sexes^14–18^, trust behavior in a mating context might likewise be differentially impacted by attractiveness and threat in men and women. We find the shortage of empirical research on investigating sex-specific effects in actual social behavior involving financial consequences to participants (e.g.,^19–26^) surprising (for examples from other domains, see^27–31^). We therefore decided in this study to systematically investigate the influence of attractiveness and threat in single men and women by applying a refined, incentivized trust paradigm with facially manipulated trustees of the other sex.

Attractiveness is one of the most important factors when it comes to selecting a potential mating partner^32,33^. Across sexes, people exhibit more prosocial behavior towards attractive than unattractive individuals (e.g., trust^34^; cooperation^35^; cooperation and punishment^36^). However, there is also evidence of sex-specific effects, with stronger attractiveness effects in other- than same-sex interactions, and in males than females^37^; but see also^32^. Evolutionary accounts like the “mate selection theory” state that men are particularly apt to approach attractive women, as attractiveness signals information about youth and good reproductive fitness, while women pay more attention to other “mating characteristics” signaling the potential ability to provide resources for future offspring^38,39^. So far, however, there is no evidence whether such sex-specific effects of attractiveness extend to the trust-behavior domain within a potential mating context. In the present study, we try to close this gap by investigating whether the facial attractiveness of other-sex individuals may modulate trust behavior displayed towards them.

Another facial feature that is key to assessing a potential partner’s trustworthiness is threat. Both men and women demonstrate lower trust levels towards individuals possessing threatening facial features, probably because threatening faces signal both harmful intentions and the capability of causing harm (i.e., dominance^11,40^). Research on the neurobiological foundations of trust further corroborates the key role of threat cues in trust behavior. Brain regions involved in detecting threat (e.g., amygdala, insula) are crucially involved in assessing another person’s trustworthiness^41,42^, and intranasal administration of the hormone oxytocin increases trust in men by inhibiting threat-related brain activity^43–45^. There seem to be interesting sex differences in these neurohormonal effects^46–48^ as well as in the general sensitivity to threat cues. Women display greater attentional bias towards threat cues, which may contribute to their higher risk for developing an anxiety disorder^49–52^. From an evolutionary perspective, abusing trust is accompanied by even higher relative costs for women due to their significantly higher parenting investments (i.e., women possess relatively few ovules and invest more time in gestation and lactation^53^ and their weaker physical strength makes them potential victims of male aggressive behavior^54–57^). Consequently, threat cues might be relatively more important for women than for men. In sum, whereas both sexes seem to lose trust in response to threatening faces, we lack empirical research on potential sex differences in these effects in other-sex interactions.

In this study, we investigated potential sex-specific effects of both attractiveness and threat on trust behavior in 93 heterosexual participants (46 women). To control for confounding changes in females’ preferences for male faces’ characteristics across the menstrual cycle^58,59^, we tested all women during their luteal phase and excluded women taking oral contraceptives. In addition to the solid evidence that women in the luteal phase are most similar to men in their stress response^60^, there is evidence for the similar processing of emotional faces of men and women during the luteal phase^61^. Participants took part in an incentivized trust game with individuals of the other sex represented by photos revealing the low- or high-intensity facial features attractiveness and threat, respectively. By using these face stimuli, our research approach aims to expand upon previous research studying incentivized social behavior in interactions between anonymous individuals (e.g.,^62–64^, and also see^65^). In line with previous findings, we hypothesized that both female and male participants would transfer more money to attractive than to unattractive individuals (= more trust), and to unthreatening compared to threatening individuals. Moreover, we aimed to examine unexplained sex differences in the relative importance of facial attractiveness and facial threat with regard to trust behavior. On the basis of the evolutionary biological assumption that sexual selection has shaped distinct strategies for assessing attractiveness and threat in males and females, we further hypothesized that threat cues would carry higher relative importance than attractiveness cues in women compared to men.

## Results

### Facial phenotypes and trust

First, we analyzed whether female and male participants trusted attractive other-sex trustees more often than unattractive ones. As expected, we detected a significant main effect of *facial attractiveness* (*F*_1, 91_ = 75.854, *p* < 0.001, *η*_*p*_^2^ = 0.455), indicating that participants generally trusted attractive faces more often. Second, we tested whether female and male participants trusted threatening faces less often than unthreatening faces. The significant main effect of *facial threat* (*F*_1, 91_ = 128.810, *p* < 0.001, *η*_*p*_^2^ = 0.586) confirmed this hypothesis. There was no significant interaction between *facial threat* and *facial attractiveness* (*F*_1, 91_ = 2.636, *p* = 0.108, *η*_*p*_^2^ = 0.028). Thus, both facial features, attractiveness and threat, revealed a significant influence on trusting behavior towards potential other-sex partners in men and women.

### Sex, facial phenotypes, and trust

**Figure 1:**
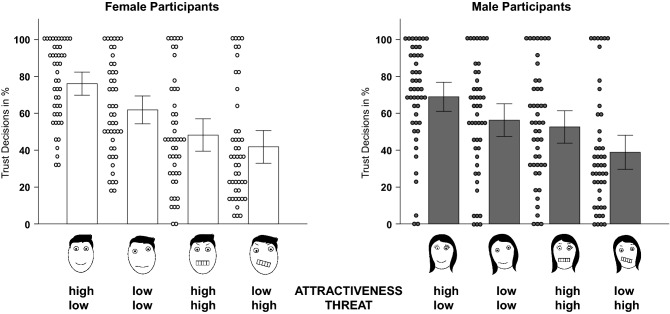
Effects of attractiveness and threat on trust behavior. Phenotypes from left to right are: ‘high attractiveness and low threat’, ‘low attractiveness and low threat’, ‘high attractiveness and high threat’, ‘low attractiveness and high threat’. Left: Trust decisions in percent for female participants. All differences are statistically significant (all *p* < 0.011). Right: Trust decisions in percent for male participants. All differences are statistically significant (all *p* < 0.001), except for the difference between the faces of the phenotypes ‘low attractiveness and low threat’ and ‘high attractiveness and high threat’. Please note that the symbolic faces in this figure represent schematic representations of the actual stimuli that were derived from photos of real human faces (for details, see Fig. 2). Bars represent standard errors; dots represent the mean trust decision in percent for each participant and each phenotype. For mean values and standard deviations of trust decisions, rating values and response times, see Tables S2–S4, respectively, in the Supplementary Material.

There was no significant main effect of sex on either trust across phenotypes (*F*_1, 91_ = 0.276, *p* = 0.601, *η*_*p*_^2^ = 0.003), or on trust for the four different phenotypes (high facial attractiveness and low facial threat: *t*_91_ = 1.415, *p* = 0.161; low facial attractiveness and low facial threat: *t*_91_ = 0.955, *p* = 0.342; high facial attractiveness and high facial threat: *t*_91_ =  − 0.706, *p* = 0.482; low facial attractiveness and high facial threat: *t*_91_ = 0.456, *p* = 0.649; see Fig. 1). To analyze sex differences in the effects of attractiveness and threat on trust behavior, we next looked at those factors’ interaction effects, and found no significant interactions between *participants’ sex* and *facial attractiveness* (*F*_1, 91_ = 1.133, *p* = 0.290, *η*_*p*_^2^ = 0.012), or between *participants’ sex* and *facial threat* (*F*_1, 91_ = 3.878, *p* = 0.052, *η*_*p*_^2^ = 0.041). However, we observed a significant interaction between *facial attractiveness* × *facial threat* × *participants’ sex* (*F*_1, 91_ = 4.562, *p* = 0.035, *η*_*p*_^2^ = 0.048). Separate ANOVAs for men and women showed that the interaction effect of *facial attractiveness* × *facial threat* was significant in women (*F*_1, 45_ = 6.097, *p* = 0.017, *η*_*p*_^2^ = 0.119), but not in men (*F*_1, 46_ = 0.155, *p* = 0.696, *η*_*p*_^2^ = 0.003). Following up on this interaction, we found that the difference in trust decisions between the dimensions *low* and *high facial attractiveness* was greater for *low threat* (∆*M* = 0.142, *SD* = 0.176, *p* < 0.001), than *high threat (∆M* = 0.064, *SD* = 0.164, *p* = 0.011). Comparing the main effects in both ANOVAs in women, we found that the influence (i.e., the effect size) of *facial threat* (*F*_1, 45_ = 79.695, *p* < 0.001, *η*_*p*_^2 ^= 0.639) was stronger than that of *facial attractiveness* (*F*_1, 45_ = 28.124, *p* < 0.001, *η*_*p*_^2 ^= 0.385; statistical comparison of effect sizes: Cohen’s *q* = 0.371; medium effect). In men, on the other hand, we found that there was no difference between the influence of *facial threat* (*F*_1, 46_ = 49.352, *p* < 0.001, *η*_*p*_^2 ^= 0.518) and *facial attractiveness* (*F*_1, 46_ = 49.646, *p* < 0.001, *η*_*p*_^2 ^= 0.519; statistical comparison of effect sizes: Cohen’s *q* = 0.001; no effect). Hence, in men, attractiveness and threat equally affected trust, while in women, threat had a stronger effect on trust than attractiveness.

### Sex, facial phenotypes, and stimulus perception

To ensure that the sex difference in the trust decisions between the phenotypes was not caused by different stimulus perceptions across female and male participants, we analyzed the ratings of attractiveness perception and threat perception. As the stimuli were rated separately for attractiveness and threat, we conducted two separate ANOVAs: one with the mean ratings for *attractiveness perception* as dependent variable, and the other with the mean ratings for *threat perception* as dependent variable. We identified no interaction between *participants’ sex* × *facial attractiveness* × *facial threat* in either ANOVA (ANOVA for rating attractiveness: *F*_1, 91_ = 1.804, *p* = 0.183, *η*_*p*_^2^ = 0.019; ANOVA for rating threat: *F*_1, 91_ = 0.421, *p* = 0.518, *η*_*p*_^2^ = 0.005; for details see Supplementary Material). In sum, there was no interaction effect of attractiveness, threat, and sex in stimulus perception, suggesting that the observed difference in trust behavior between female and male participants was not caused by different attractiveness or threat perceptions in the two groups.

## Discussion

In this study, we investigated the sex-specific effects of the facial features attractiveness and threat on trusting individuals of the other sex. Using a trust game with actual financial consequences for participants, we systematically varied the intensities of facial attractiveness and facial threat of other-sex trustees. We found that both male and female participants exhibited more trust towards attractive and unthreatening-looking potential partners. However, the relative importance of these two facial features differed in men and women: compared to men’s behavior, women’s trust behavior was more strongly influenced by a threatening-looking than by an attractive-looking individual. Importantly, this difference in social behavior was not driven by a sex difference in social perception, suggesting that women value threat more than attractiveness in their trust decisions than do men, even though their perception of these features is similar.

By experimentally modulating both facial attractiveness and threat, our results expand upon earlier studies that investigated the effects of these features in social interactions separately from one another. In line with previous research, we detected main effects of attractiveness (e.g.^34^) and threat (e.g.^11^) on trust in both sexes. We failed to observe that these effects differed in strength across sexes (as some empirical and theoretical research has suggested, e.g.^37,50^). However, a sex difference did emerge in the relative importance of both features, with women in their luteal phase placing more emphasis on threat than on attractiveness, and men valuing both features equally. More specifically, women trusted attractive but threatening-looking trustees less than unattractive but unthreatening ones, whereas men demonstrated indistinguishable levels of trust towards both phenotypes. In other words, a partner’s high attractiveness could “compensate for” high threat in male but not female participants. From an evolutionary perspective, being relatively more wary of potential threat than vulnerable to attractiveness cues in a mating context might be a highly adaptive mechanism for women, because having one’s trust abused is more costly, and approaching attractive men of “good health” is less beneficial for them^66–68^. Moreover, empirically, men (but not women) given face cues related to threat (i.e., greater facial width) abuse trust more often^3^ and (young) men perceived as more attractive by women reveal lower degrees of prosocial behavior^69,70^. Although the accuracy of social inferences from facial cues remains equivocal and people certainly refine their quick facial assessments of another’s trustworthiness on the basis of other cues and experiences during an interaction^71^, these findings suggest that it benefits women to prioritize threat over attractiveness in trust decisions that rely on the partner’s facial appearance.

When searching for explanations for this sex difference we identified in the relative importance of attractiveness and threat on trust, it is important to keep in mind that none of the women in this study were taking hormonal contraceptives, and that they were participating in this experiment during their menstrual cycle’s luteal phase. This phase, which starts after ovulation, is characterized by high levels of progesterone and to a lesser extent estradiol^72,73^. Interestingly, high levels of both hormones are known to be associated with enhanced sensitivity to threat-related facial cues in women^74,75^. As the highest levels of these hormones are observed during the mid-luteal phase when, potentially, conception has already taken place, and during pregnancy^76^, one could speculate that these steroids trigger behavior that protects fetal development (for a similar argument; see^46^). In a similar vein, the “dual sexuality” hypothesis argues that women may prefer sexually attractive men on days before ovulation when they are looking for conception partners, whereas they prefer men who appear unthreatening during their luteal phase when they are seeking high-investing partners with father qualities^77–79^. Indeed, our study provides evidence that women in their luteal phase are more cautious than men, or less willing, to trust potentially threatening but attractive partners, perhaps because the price of having one’s trust abused is particularly high during this phase of the menstrual cycle when pregnancy is possible. Following the dual sexuality hypothesis, one could speculate that facial attractiveness might become more important for women when making trust decisions during the days before ovulation. Future research could systematically test this hypothesis (see^80^, for example).

So far, we have interpreted the present study’s findings as evidence demonstrating that male and female participants evaluate attractiveness and threat differentially in trusting other-sex individuals (= participant’s sex effect). However, one could also argue that our findings demonstrate that male and female faces are evaluated differently (= face stimulus’s sex effect). As the present study investigates approach behavior in other-sex interactions, female participants always interacted with male-face stimuli, and male participants always interacted with female-face stimuli, rendering it difficult to disentangle the participant’s sex from the stimulus’ sex effect. Interestingly, research on trait judgments from faces has revealed that judgments of female in comparison to male faces are less differentiated with regard to the effects of distinct facial characteristics and more based on a general impression of the face’s valence^81^. Transferring these findings to the present study, male participants did not distinguish between faces signaling ambiguous information about trustworthiness (trust level: unattractive and unthreatening = attractive and threatening), because they might evaluate ambiguous female faces less differentiatedly and assign intermediate valence to such faces. In contrast, female participants, who distinguished between faces offering ambiguous information (trust level: unattractive and unthreatening > attractive and threatening), might evaluate facial attractiveness and threat independently from one another, leading to a more differentiated assessment of trustworthiness towards ambiguous faces. To better distinguish the effects of participants’ and stimulus’ sex, future studies could investigate both same-sex and other-sex interactions.

In sum, the present study provides first evidence of a sex-specific interacting effect of a potential partner’s facial attractiveness and threat on trust behavior. From an evolutionary perspective, the fact that women in their luteal phase prioritize threat over attractiveness might be explained by their higher parental investment and the high price of being abused when they might get pregnant^82^. Future studies might include women in their pre-ovulatory phase (validated by blood assays to measure gonadal steroid) to observe whether women do indeed prioritize attractiveness over threat during this menstrual phase, thereby contributing to the ongoing debate about whether women’s preferences for their partner’s attractiveness and threat change across the menstrual cycle^83,84^. Furthermore, we expect our findings to stimulate research refining such explanations by illuminating the possible role of non-evolutionary explanations for sex differences (e.g., sex stereotypes^85^, societal influences^86^), or by revealing the role of individual differences in traits (e.g., general dispositional trust^87^; attachment styles^88^) or states (e.g., recent trust-related experiences such as trust violations^89^) and their interaction with sex effects in affecting trust in other-sex interactions. It could also prove fascinating to investigate the effects of attractiveness and threat in couples in both intra- and extra-couple interactions and in homosexual male and female couples (e.g.^90^), as well as non-mating contexts like approaching new potential friends. Overall, this research may help us better understand which factors impact trust as a fundamental ingredient in intimate social relationships.

## Methods

### Participants

We recruited a sample of 93 healthy participants (47 men, 46 women; age: *M* = 22.44, *SD* = 3.85, range: 18–34). All participants were single (i.e., not in a couple relationship^91^) and attracted to the other sex (rated five or higher on a 7-point Likert scale from ‘not at all’ [1] to ‘absolutely’ [7]). Exclusion criteria were alcohol, nicotine or drug abuse, studying or having a degree in psychology or economics, current or previous history of psychiatric disorders, or insufficient fluency in the German language. All female participants took part in the experiment during their menstrual cycle’s luteal phase^59,60^ and were free of hormonal contraceptives. The luteal phase was determined by self-report: the earliest testing day was determined by adding half of the maximum cycle length duration, plus a 2-day buffer to the first day of menstruation, and to determine the latest testing day we added the minimal cycle length duration to the first day of menstruation (see Supplementary Material for further details). The Ethics Committee of the University of Freiburg approved this study, which was conducted according to the principles expressed in the Declaration of Helsinki.

### Procedure

We recruited participants using flyers and bulletins. Participants had to fill out an online screening to assess exclusion criteria^92^. We telephoned eligible participants to offer appointments for the experimental session, which took place in a group laboratory with up to 12 participants taking part simultaneously. Participants provided written informed consent to participate in the study and have their photo taken. For the picture displayed in Fig. 3B, we furthermore obtained informed consent of that participant for publication of identifying images. To reduce demand characteristics, we included a cover story in the study. We told participants that their photos would be utilized in the trust game paradigm (see below) to ensure that participants believed that they would later be interacting with real participants. Importantly, none of the participants expressed any disbelief about the genuineness of the trust game after the experiment. To keep the photos taken in our laboratory comparable to the final set of face stimuli taken from the Basel Face Database^93^, participants were sent into another room set up for professional photography. All participants wore a black T-Shirt and were instructed to look into the camera with a neutral expression. Afterwards, participants received instructions for the trust game and performed practice trials. Participants played 88 rounds of the trust game, each with a different other-sex individual represented by a color photo, followed by a stimuli perception paradigm, in which participants rated the photos they saw in the trust game for attractiveness and threat. Both paradigms took 10–15 min. At the end of the study, participants received their reimbursement of 15 Euros basic payment plus on average 7.03 Euros additional payoff for the trust game (*SD* = 0.566, range = 5.02–8.70; for details, see below).

### Face stimuli

**Figure 2:**
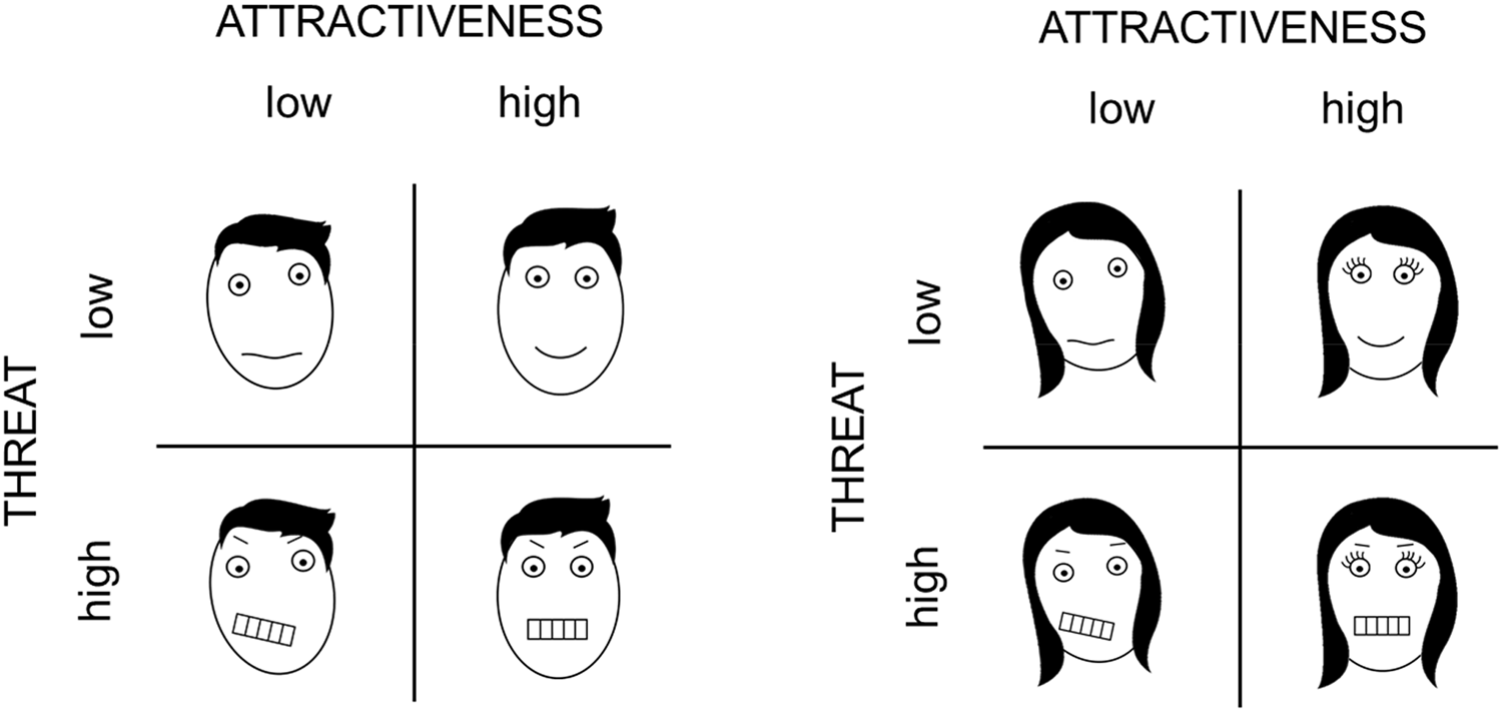
Drawings of representative male (left) and female stimulus set (right). There were four different phenotypes with 22 faces each revealing either *low-* or *high*-intensity ‘attractiveness’ and ‘threat’. In total, every set contained 88 faces, each generated from a unique face identity. The symbolic faces in this figure represent prototypes by which to illustrate the face phenotypes, facial symmetry codes attractiveness (asymmetric = low attractiveness), and mouth codes threat (showing teeth = high threat). Please note that this figure illustrates schematic representations of the stimulus categories for visualization purposes, and that the actual stimuli were derived from photos of real human faces exhibiting neutral expressions (i.e., displaying no teeth or other emotionally-loaded expressions; see Fig. 3B for a stimulus example).

We created two sets of stimuli: one with female faces that were presented to male participants, and one with male faces presented to female participants. We strictly used neutral faces not demonstrating any emotional states. Faces were manipulated to reveal two features of low or high intensity, namely attractiveness and threat (e.g., ‘high threat and low attractiveness’), resulting in four phenotypes (see Fig. 2). Faces were taken from the Basel Face Database^93^, as well as from pictures shot by our own photographer. Face stimuli were generated by applying a data-driven, computational approach that captured the variance in facial structure that signals specific social attributions^42,94^. This approach enabled us to parametrically manipulate the values of facial attractiveness and threat based on previously collected other-sex ratings. Based on those ratings, vectors were created and applied to the photographed faces with a neutral facial expression, which caused faces to have high or low values on attractiveness and high or low values threat (e.g., ‘high threat’ and ‘low attractiveness’; also see^95,96^). These newly-created faces were then rated by 53 participants (20 women, 33 men, age: *M* = 24.17, *SD* = 3.57) in a pilot study. Afterwards, we selected the 22 distinct faces most characteristic of each phenotype, resulting in 88 faces with subtle attractiveness and threat manipulations per stimulus set so that the phenotype should not have been apparent to the participants. As required for the purpose of this experiment, all *t*-tests comparing threat and attractiveness ratings of faces with the same intensity (i.e., low vs. low or high vs. high) were not significant (*p* > 0.078), whereas all *t*-tests comparing threat and attractiveness ratings of faces with different intensity (i.e., low vs. high) were significant (*p* < 0.001; for details, see Supplementary Material).

### Trust game paradigm

**Figure 3:**
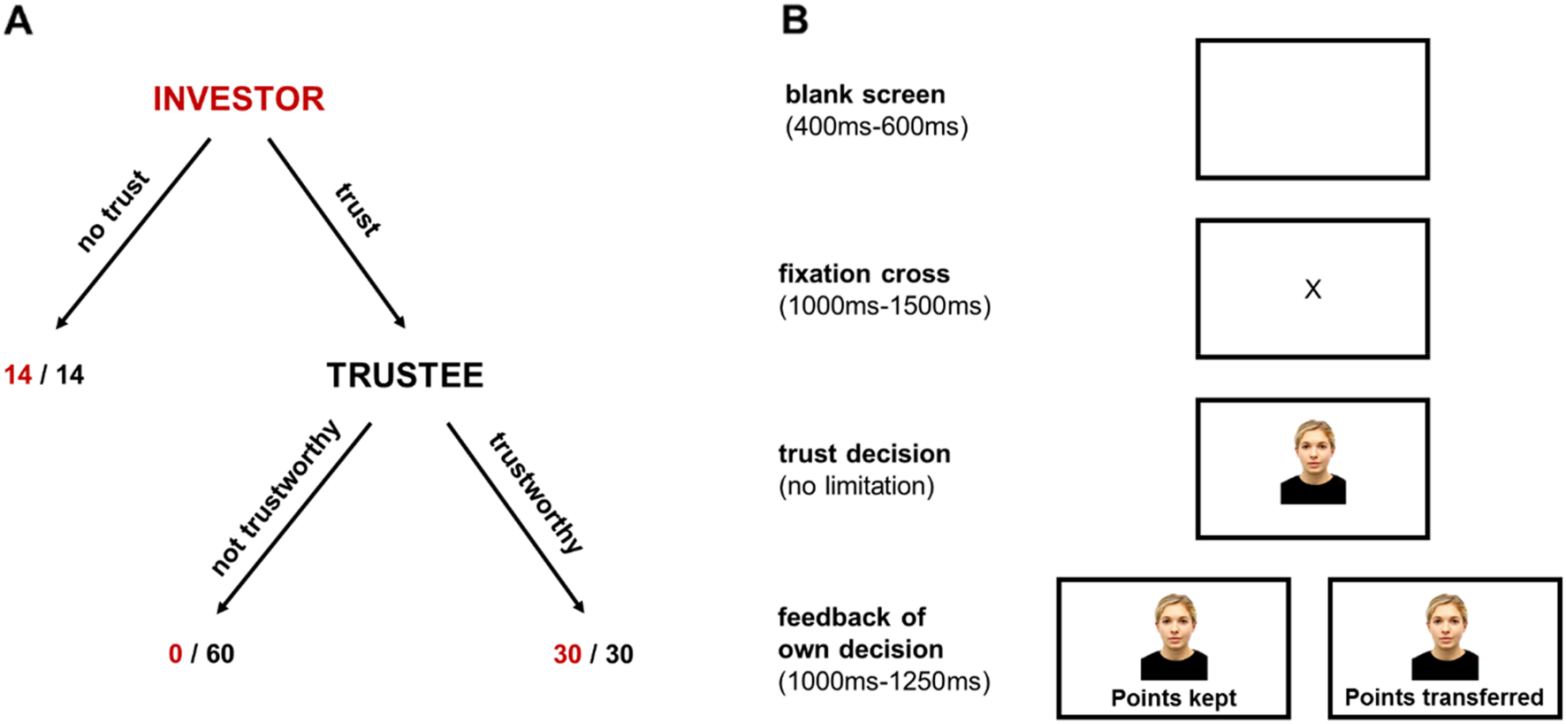
Modified version of the trust game. (**A**) Payoff structure. Participants were assigned the role of the investor. If the investor decided not to trust the trustee, both investor and trustee received 14 MUs. If the investor decided to trust, the trustee could either keep everything for him/herself (0/60) or transfer half of the points back to the investor (30/30). (**B**) Example trial with screen durations in milliseconds (ms). First, participants saw a blank screen for 400–600 ms, followed by fixation cross for 1000–1500 ms. Then, participants saw a photo of the trustee until they made their decision, which was then displayed for 1000–1250 ms.

Participants took part in a slightly modified version of the trust game^97,98^ (for details on screen presentation durations, see Fig. 3). Participants were assigned the role of the investor, and, in each round, saw a face from the other sex’s stimulus set representing the trustee. Both investor and trustee received an initial endowment to ensure that no social preference other than trust (e.g., inequality aversion, altruism) would influence the participant’s decision about whether to transfer money to the trustee. Participants could then decide whether to keep their endowment, in which case both the investor and trustee received 14 MU, or to transfer their endowment. If they transferred their endowment, the trustee could either keep everything for him/herself (‘not trustworthy’), in which case the investor received 0 MU and the trustee received 60 MU, or transfer back half of it (‘trustworthy’), in which case both the investor and trustee received 30 MU. We chose a payoff structure (see Fig. 3) which triggered trust-decision rates of approximately 50% in previous studies without face stimuli^99^, thus enabling both the up- and down-modulation of trust decision rates by varying facial features. At the end of the study, participants received their payoff according to the following exchange ratio: 100 MU = 0.50€. Participants were told that a subgroup of participants had been randomly selected as trustees who had already made their decision whether to return money. In fact, to determine payments, we randomly drew trustee decisions from a distribution of decisions collected during our laboratory’s previous studies^43^. Participants learned about trustees’ decisions at the end of the experiment to avoid any influence of reciprocal or reputational considerations of previous interactions on trust behavior. This paradigm was programmed using Presentation software (Version 18.0, Neurobehavioral Systems, Inc., Berkeley, CA).

### Stimuli perception paradigm

Participants rated all 88 faces for attractiveness and threat, respectively, on a 7-point Likert scale from ‘very unattractive’/‘very unthreatening’ to ‘very attractive’/‘very threatening’. The presentation order of the photos was randomized. In total, there were four blocks with 44 photos, respectively. At the beginning of each block, participants learned which facial feature they should rate (attractiveness or threat). Whether participants rated attractiveness or threat in block one and three was also randomized, and followed by the other feature in the subsequent block (i.e., block two and four, respectively).

### Statistical analysis

All analyses were conducted with the software SPSS (27th edition). We first calculated the mean trust for each phenotype by dividing the number of decisions to trust a specific phenotype by 22, i.e., the total number of trust decisions for each phenotype. To test the influence of the facial features attractiveness and threat on mean trust, and whether there were any differences between men and women in the trust decisions, we conducted a repeated-measures ANOVA with *participants’ sex* (women and men) as a between-subject factor, and *facial attractiveness* (low vs. high) and *facial threat* (low vs. high) as within-subject factor. We then compared the effect sizes of the main effects *facial attractiveness* and *facial threat* to investigate any sex differences in the importance of both features. For that purpose, we transformed the *η*_*p*_^2^ effect size of each main effect to the effect size *r* and then compared those within the sexes^100,101^.

The Stimuli Perception Paradigm was used to show that any differences in the trust game decisions between sexes were not due to differing attractiveness or threat perceptions of the distinct other-sex stimulus sets. We first calculated mean ratings for facial attractiveness perception and facial threat perception by averaging the ratings of the 22 faces of each phenotype. Then, we conducted two repeated measures ANOVAs, one for the mean ratings of *attractiveness perception* and one for the mean ratings of *threat perception* as dependent variable. We included *participants’ sex* (women and men) as a between-subject factor, and *facial attractiveness* (low vs. high) and *facial threat* (low vs. high) as within-subject factors.

## Supplementary Information

Supplementary Information.

## References

[1] Balliet D, Van Lange PAM (2013). Trust, conflict, and cooperation: A meta-analysis. Psychol. Bull..

[2] Fletcher GJO, Simpson JA, Thomas G (2000). The measurement of perceived relationship quality components: A confirmatory factor analytic approach. Pers. Soc. Psychol. Bull..

[3] Stirrat M, Perrett DI (2010). Valid facial cues to cooperation and trust: Male facial width and trustworthiness. Psychol. Sci..

[4] Holt-Lunstad J, Smith TB, Layton JB (2010). Social relationships and mortality risk: A meta-analytic review. PLoS Med..

[5] Kleinert T (2020). The Trust Game for Couples (TGC): A new standardized paradigm to assess trust in romantic relationships. PLoS ONE.

[6] Rempel JK, Holmes JG, Zanna MP (1985). Trust in close relationships. J. Pers. Soc. Psychol..

[7] Frith CD (1999). Interacting minds—A biological basis. Science.

[8] van Wout M, Sanfey AG (2008). Friend or foe: The effect of implicit trustworthiness judgments in social decision-making. Cognition.

[9] Little AC, Jones BC, DeBruine LM (2011). Facial attractiveness: Evolutionary based research. Philos. Trans. R. Soc. B Biol. Sci..

[10] Maner JK (2003). Sexually selective cognition: Beauty captures the mind of the beholder. J. Pers. Soc. Psychol..

[11] Oosterhof NN, Todorov A (2008). The functional basis of face evaluation. Proc. Natl. Acad. Sci..

[12] Puts DA, Jones BC, DeBruine LM (2012). Sexual selection on human faces and voices. J. Sex Res..

[13] Thornhill R, Gangestad SW (1993). Human facial beauty: Averageness, symmetry, and parasite resistance. Hum. Nat..

[14] Buss DM (1999). Evolutionary Psychology: The New Science of the Mind.

[15] Puts DA (2010). Beauty and the beast: Mechanisms of sexual selection in humans. Evol. Hum. Behav..

[16] Shuster SM (2009). Sexual selection and mating systems. Proc. Natl. Acad. Sci..

[17] Kleisner K (2021). How and why patterns of sexual dimorphism in human faces vary across the world. Sci. Rep..

[18] Tognetti A, Dubois D, Faurie C, Willinger M (2016). Men increase contributions to a public good when under sexual competition. Sci. Rep..

[19] Gianotti LRR, Nash K, Baumgartner T, Dahinden FM, Knoch D (2018). Neural signatures of different behavioral types in fairness norm compliance. Sci. Rep..

[20] Nash K, Leota J, Tran A (2021). Neural processes in antecedent anxiety modulate risk-taking behavior. Sci. Rep..

[21] Schiller B, Domes G, Heinrichs M (2020). Oxytocin changes behavior and spatio-temporal brain dynamics underlying inter-group conflict in humans. Eur. Neuropsychopharmacol..

[22] Schiller B, Baumgartner T, Knoch D (2014). Intergroup bias in third-party punishment stems from both ingroup favoritism and outgroup discrimination. Evol. Hum. Behav..

[23] Schiller B, Kleinert T, Teige-Mocigemba S, Klauer KC, Heinrichs M (2020). Temporal dynamics of resting EEG networks are associated with prosociality. Sci. Rep..

[24] Nash K, Schiller B, Gianotti LRR, Baumgartner T, Knoch D (2013). Electrophysiological indices of response inhibition in a Go/NoGo task predict self-control in a social context. PLoS ONE.

[25] Nash K, Gianotti LRR, Knoch D (2015). A neural trait approach to exploring individual differences in social preferences. Front. Behav. Neurosci..

[26] Tusche A, Böckler A, Kanske P, Trautwein F-M, Singer T (2016). Decoding the charitable brain: Empathy, perspective taking, and attention shifts differentially predict altruistic giving. J. Neurosci. Off. J. Soc. Neurosci..

[27] McCarthy MM, Woolley CS, Arnold AP (2017). Incorporating sex as a biological variable in neuroscience: What do we gain?. Nat. Rev. Neurosci..

[28] Wu Y, Zhang Y, Ou J, Hu Y, Zilioli S (2020). Exogenous testosterone increases the audience effect in healthy males: Evidence for the social status hypothesis. Proc. Biol. Sci..

[29] Santos LR, Rosati AG (2015). The evolutionary roots of human decision making. Annu. Rev. Psychol..

[30] House BR, Henrich J, Brosnan SF, Silk JB (2012). The ontogeny of human prosociality: Behavioral experiments with children aged 3 to 8. Evol. Hum. Behav..

[31] Wuttke-Linnemann A, Nater UM, Ehlert U, Ditzen B (2019). Sex-specific effects of music listening on couples’ stress in everyday life. Sci. Rep..

[32] Eastwick PW, Luchies LB, Finkel EJ, Hunt LL (2014). The predictive validity of ideal partner preferences: A review and meta-analysis. Psychol. Bull..

[33] Lobmaier JS, Fischbacher U, Wirthmüller U, Knoch D (2018). The scent of attractiveness: Levels of reproductive hormones explain individual differences in women’s body odour. Proc. Biol. Sci..

[34] Wilson RK, Eckel CC (2006). Judging a book by its cover: Beauty and expectations in the trust game. Polit. Res. Q..

[35] Farrelly D, Lazarus J, Roberts G (2007). Altruists attract. Evol. Psychol..

[36] Lucas M, Koff E (2013). How conception risk affects competition and cooperation with attractive women and men. Evol. Hum. Behav..

[37] Maestripieri D, Henry A, Nickels N (2017). Explaining financial and prosocial biases in favor of attractive people: Interdisciplinary perspectives from economics, social psychology, and evolutionary psychology. Behav. Brain Sci..

[38] Buss DM, Schmitt DP (1993). Sexual strategies theory: An evolutionary perspective on human mating. Psychol. Rev..

[39] Farrelly D, Clemson P, Guthrie M (2016). Are women’s mate preferences for altruism also influenced by physical attractiveness?. Evol. Psychol..

[40] Campellone TR, Kring AM (2013). Who do you trust? The impact of facial emotion and behaviour on decision making. Cogn. Emot..

[41] Declerck CH, Boone C, Emonds G (2013). When do people cooperate? The neuroeconomics of prosocial decision making. Brain Cogn..

[42] Todorov A, Baron SG, Oosterhof NN (2008). Evaluating face trustworthiness: A model based approach. Soc. Cogn. Affect. Neurosci..

[43] Baumgartner T, Heinrichs M, Vonlanthen A, Fischbacher U, Fehr E (2008). Oxytocin shapes the neural circuitry of trust and trust adaptation in humans. Neuron.

[44] Kosfeld M, Heinrichs M, Zak PJ, Fischbacher U, Fehr E (2005). Oxytocin increases trust in humans. Nature.

[45] Schiller B, Heinrichs M, Heinrichs M, Schultheiss OC, Mehta PH (2018). The neuroendocrinological basis of human affiliation: How oxytocin coordinates affiliation-related cognition and behavior via changing underlying brain activity. Routledge International Handbook of Social Neuroendocrinology.

[46] Domes G (2010). Effects of intranasal oxytocin on emotional face processing in women. Psychoneuroendocrinology.

[47] Spengler FB (2017). Kinetics and dose dependency of intranasal oxytocin effects on amygdala reactivity. Biol. Psychiatry.

[48] Ditzen B (2013). Sex-specific effects of intranasal oxytocin on autonomic nervous system and emotional responses to couple conflict. Soc. Cogn. Affect. Neurosci..

[49] Catuzzi JE, Beck KD (2014). Anxiety vulnerability in women: a two-hit hypothesis. Exp. Neurol..

[50] Hampson E, van Anders SM, Mullin LI (2006). A female advantage in the recognition of emotional facial expressions: Test of an evolutionary hypothesis. Evol. Hum. Behav..

[51] McClure EB (2004). A developmental examination of gender differences in brain engagement during evaluation of threat. Biol. Psychiatry.

[52] Remes O, Brayne C, van der Linde R, Lafortune L (2016). A systematic review of reviews on the prevalence of anxiety disorders in adult populations. Brain Behav..

[53] Trivers RL (1996). Parental Investment and Sexual Selection.

[54] Benenson JF (2013). The development of human female competition: Allies and adversaries. Philos. Trans. R. Soc. Lond. B. Biol. Sci..

[55] Ein-Dor T, Perry-Paldi A, Hirschberger G, Birnbaum GE, Deutsch D (2015). Coping with mate poaching: Gender differences in detection of infidelity-related threats. Evol. Hum. Behav..

[56] Lindenfors P, Tullberg BS (2011). Evolutionary aspects of aggression the importance of sexual selection. Adv. Genet..

[57] Nivette A, Sutherland A, Eisner M, Murray J (2019). Sex differences in adolescent physical aggression: Evidence from sixty-three low-and middle-income countries. Aggress. Behav..

[58] Penton-Voak IS (1999). Menstrual cycle alters face preference. Nature.

[59] Penton-Voak IS, Perrett DI (2000). Female preference for male faces changes cyclically: Further evidence. Evol. Hum. Behav..

[60] Kirschbaum C, Kudielka BM, Gaab J, Schommer NC, Hellhammer DH (1999). Impact of gender, menstrual cycle phase, and oral contraceptives on the activity of the hypothalamus-pituitary-adrenal axis. Psychosom. Med..

[61] Guapo VG (2009). Effects of sex hormonal levels and phases of the menstrual cycle in the processing of emotional faces. Psychoneuroendocrinology.

[62] Camerer CF (2011). Behavioral Game Theory: Experiments in Strategic Interaction.

[63] Manski CF (2000). Economic analysis of social interactions. J. Econ. Perspect..

[64] Camerer CF, Fehr E (2006). When does ‘economic man’ dominate social behavior?. Science.

[65] Vick S-J, Waller BM, Parr LA, Smith Pasqualini MC, Bard KA (2007). A cross-species comparison of facial morphology and movement in humans and chimpanzees using the facial action coding system (FACS). J. Nonverbal Behav..

[66] Bjorklund DF, Shackelford TK (1999). Differences in parental investment contribute to important differences between men and women. Curr. Dir. Psychol. Sci..

[67] Buss DM (2007). The evolution of human mating. Acta Psychol. Sin..

[68] Todd PM, Penke L, Fasolo B, Lenton AP (2007). Different cognitive processes underlie human mate choices and mate preferences. Proc. Natl. Acad. Sci..

[69] Price ME, Brown S, Dukes A, Kang J (2015). Bodily attractiveness and egalitarianism are negatively related in males. Evol. Psychol..

[70] Shinada M, Yamagishi T (2014). Physical attractiveness and cooperation in a prisoner’s dilemma game. Evol. Hum. Behav..

[71] Todorov A, Olivola CY, Dotsch R, Mende-Siedlecki P (2015). Social attributions from faces: Determinants, consequences, accuracy, and functional significance. Annu. Rev. Psychol..

[72] Hawkins SM, Matzuk MM (2008). The menstrual cycle: Basic biology. Ann. N. Y. Acad. Sci..

[73] Mihm M, Gangooly S, Muttukrishna S (2011). The normal menstrual cycle in women. Anim. Reprod. Sci..

[74] Conway CA (2007). Salience of emotional displays of danger and contagion in faces is enhanced when progesterone levels are raised. Horm. Behav..

[75] Pearson R, Lewis MB (2005). Fear recognition across the menstrual cycle. Horm. Behav..

[76] Hahn-Holbrook J, Holbrook C, Schultheiss OC, Mehta PH (2018). The social neuroendocrinology of pregnancy and breastfeeding in mothers (and others). Routledge International Handbook of Social Neuroendocrinology.

[77] Grebe NM, Emery Thompson M, Gangestad SW (2016). Hormonal predictors of women’s extra-pair vs in-pair sexual attraction in natural cycles: Implications for extended sexuality. Horm. Behav..

[78] Roney, J. R. Functional roles of gonadal hormones in human pair bonding and sexuality. In *Routledge Int. Handb. Soc. Neuroendocrinol.* 239–255 (Routledge, 2018).

[79] Thornhill R, Gangestad SW (2008). The Evolutionary Biology of Human Female Sexuality.

[80] Ball A (2013). Variability in ratings of trustworthiness across the menstrual cycle. Biol. Psychol..

[81] Oh D, Dotsch R, Porter J, Todorov A (2020). Gender biases in impressions from faces: Empirical studies and computational models. J. Exp. Psychol. Gen..

[82] McNamara JM, Wolf M (2015). Sexual conflict over parental care promotes the evolution of sex differences in care and the ability to care. Proc. Biol. Sci..

[83] Gildersleeve K, Haselton MG, Fales MR (2014). Do women’s mate preferences change across the ovulatory cycle? A meta-analytic review. Psychol. Bull..

[84] Wood W, Kressel L, Joshi PD, Louie B (2014). Meta-analysis of menstrual cycle effects on women’s mate preferences. Emot. Rev..

[85] Spencer SJ, Steele CM, Quinn DM (1999). Stereotype threat and women’s math performance. J. Exp. Soc. Psychol..

[86] Ridgeway CL, Correll SJ (2004). Unpacking the gender system: A theoretical perspective on gender beliefs and social relations. Gend. Soc..

[87] Evans A, Zeigler-Hill V, Shackelford TK (2016). Interpersonal trust scale. Encyclopedia of Personality and Individual Differences.

[88] Ainsworth MS, Bowlby J (1991). An ethological approach to personality development. Am. Psychol..

[89] Mikulincer M (1998). Attachment working models and the sense of trust: An exploration of interaction goals and affect regulation. J. Pers. Soc. Psychol..

[90] Appleby PR, Miller LC, Rothspan S (1999). The paradox of trust for male couples: When risking is a part of loving. Pers. Relatsh..

[91] Little AC, Jones BC, Penton-Voak IS, Burt DM, Perrett DI (2002). Partnership status and the temporal context of relationships influence human female preferences for sexual dimorphism in male face shape. Proc. R. Soc. Lond. B Biol. Sci..

[92] Schiller, B., Tönsing, D., Kleinert, T., Böhm, R. & Heinrichs, M. Effects of the COVID-19 pandemic nationwide lockdown on mental health, environmental concern, and prejudice against other social groups. *Environ. Behav.*, 1–12. 10.1177/001391652110365211036991 (2018).

[93] Walker M, Schönborn S, Greifeneder R, Vetter T (2018). The Basel face database: A validated set of photographs reflecting systematic differences in big two and big five personality dimensions. PLoS ONE.

[94] Walker M, Vetter T (2016). Changing the personality of a face: Perceived big two and big five personality factors modeled in real photographs. J. Pers. Soc. Psychol..

[95] Rudert SC, Reutner L, Greifeneder R, Walker M (2017). Faced with exclusion: Perceived facial warmth and competence influence moral judgments of social exclusion. J. Exp. Soc. Psychol..

[96] Todorov A, Dotsch R, Porter JM, Oosterhof NN, Falvello VB (2013). Validation of data-driven computational models of social perception of faces. Emotion.

[97] Berg J, Dickhaut J, McCabe K (1995). Trust, reciprocity, and social history. Games Econ. Behav..

[98] Kumar A, Capraro V, Perc M (2020). The evolution of trust and trustworthiness. J. R. Soc. Interface.

[99] von Dawans B, Fischbacher U, Kirschbaum C, Fehr E, Heinrichs M (2012). The social dimension of stress reactivity: Acute stress increases prosocial behavior in humans. Psychol. Sci..

[100] Cohen J (2013). Statistical Power Analysis for the Behavioral Sciences.

[101] Lenhard W, Lenhard A (2016). Computation of Effect Sizes.

