# Supplementary material for: Facial threat affects trust more strongly than facial attractiveness in women than it does in men
Source: Sci Rep. 2021 Nov 18;11:22475. doi: 10.1038/s41598-021-01775-5 (PMC8602253; doi:10.1038/s41598-021-01775-5)
Supplement: Supplementary file 1 — Supplementary Information. [file 41598_2021_1775_MOESM1_ESM.docx]

Supplementary Material

Facial threat affects trust more strongly than facial attractiveness in women than it does in men

Johanna Brustkern^1^, Markus Heinrichs^1^, Mirella Walker^2^, Bastian Schiller^1, *^

^1^ Laboratory for Biological and Personality Psychology, Department of Psychology, University of Freiburg, Stefan-Meier-Str. 8, 79104 Freiburg, Germany

^2^ Faculty of Psychology, University of Basel, Petersplatz 1, 4001 Basel, Switzerland

* Corresponding author.

Email: [bastian.schiller@psychologie.uni-freiburg.de](mailto:bastian.schiller@psychologie.uni-freiburg.de) (B. Schiller)

**Supplementary Material**

**Calculation of the luteal phase**

For female participants, we assessed the luteal phase by self-report. The beginning of the luteal phase was calculated in the following way: $first day of menstruation+ \frac{maximal cycle length}{2}+2$. We added these two additional days as buffer to determine the earliest possible testing day, in order to ensure that participants not take part during ovulation. The latest possible testing day was: $first day of menstruation+minimal cycle length$. We furthermore asked participants on their testing day whether they had started menstruating, and excluded them if they said yes.

**Pilot study – Stimuli selection**

The purpose of the pilot study was to find faces offering a good fit for each of the four phenotypes, hence faces with either low or a highly intensive attractiveness or threat. For the female stimuli set (presented to male participants), we had 121 identities (i.e., different faces), that were manipulated on the facial features attractiveness and threat using the Basel Face Model ^1^. This resulted in 121 identities * 4 phenotypes = 484 faces for the female stimuli set. For the male stimuli set (presented to female participants), we had 110 identities, resulting in 110 identities * 4 phenotypes = 440 faces. To select the best faces matching each phenotype, all faces were rated by the other sex for attractiveness and threat on a 7-point Likert scale from ‘very unattractive’/‘very non-threatening’ to ‘very attractive’/‘very threatening’. For this pilot study, we recruited 33 male participants and 20 female participants (age: *M* = 24.17, *SD* = 3.57) with the same inclusion criteria as the main study. The pilot study was conducted in our group laboratory with up to 12 participants of the same sex taking part simultaneously. After participants gave written informed consent, they rated the faces in blocks for either attractiveness or threat for about 60 minutes. Each block contained 44 faces in the female stimuli set and 55 faces in the male stimuli set. Faces were pseudorandomized to ensure that the same identity not appear twice within the same block, and the same phenotype (e.g., ‘high attractiveness & low threat’) not appear more than three consecutive times. Participants rated either attractiveness or threat in each block, in a pseudorandomized order with the same rating feature appearing no more than twice consecutively. Each face’s mean ratings were compared, and the best fitting faces were chosen for each phenotype. This resulted in 22 faces for each phenotype and 88 faces (all different identities) in total. Mean ratings and standard deviations are shown in Table S1. Ratings of the same feature and same intensity, e.g., ‘high attractiveness’, did not significantly differ between two phenotypes, e.g. ‘high attractiveness & low threat’ and ‘high attractiveness & high threat’, within men and women (*p* > 0.078). Ratings of the same feature but of different intensity, e.g. ‘low attractiveness’ vs. ‘high attractiveness’, differed significantly among men and women (*p* < 0.001).

| Attractiveness ratings and threat ratings | | high attractiveness &  low threat | low  attractiveness &  low threat | high attractiveness &  high threat | low attractiveness &  high threat |
| --- | --- | --- | --- | --- | --- |
| Women  *N*= 20 | Attractiveness | *M* = 3.79  *SD* = 0.94 | *M* = 2.49  *SD* = 0.79 | *M* = 3.75  *SD* = 0.84 | *M* = 2.47  *SD* = 0.71 |
|  | Threat | *M* = 2.50  *SD* = 0.84 | *M* = 2.50  *SD* = 0.80 | *M* = 3.85  *SD* = 1.28 | *M* = 3.92  *SD* = 1.25 |
| Men  *N*= 33 | Attractiveness | *M* = 4.05  *SD* = 0.85 | *M* = 2.96  *SD* = 0.82 | *M* = 3.91  *SD* = 0.81 | *M* = 2.87  *SD* = 0.83 |
|  | Threat | *M* = 3.02  *SD* = 0.83 | *M* = 3.08  *SD* = 0.85 | *M* = 3.83  *SD* = 0.95 | *M* = 3.97  *SD* = 0.99 |

Table S1: Mean values and standard deviation for attractiveness and threat ratings for the selected 88 stimuli gathered in the pilot study. Twenty women rated male stimuli and 33 men rated female stimuli. All *t*-tests for ratings of low vs. high intensities for a given feature were significant (*p* < 0.001) within each sex, and all *t*-tests for ratings low vs. low intensity or high vs. high intensity of a feature were not significant within each sex (*p* > 0.078).

**Results – Sex, Facial Phenotypes, and Stimuli Perception**

To ensure that the difference in the trust decisions between the phenotypes was not caused by different stimulus perceptions across female and male participants, we included a Stimuli Perception Paradigm. We conducted two ANOVAs, one for attractiveness ratings and one for threat ratings (see Table S2 for rating values). The ANOVA for attractiveness ratings showed a significant main effect of *attractiveness* (*F_1, 91_* = 519.084, *p*< 0.001, *η_p_*^2^= 0.851). Hence, participants rated faces with high attractiveness intensity as more attractive than faces with low attractiveness intensity. We furthermore noted a significant main effect of sex (*F_1, 91_* = 9.124, *p*= 0.003, *η_p_*^2^= 0.091). Men delivered significantly higher attractiveness ratings than women. There was no significant interaction of *attractiveness*sex* (*F_1, 91_* = 1.172, *p*= 0.282, *η_p_*^2^= 0.013), but a significant interaction of *attractiveness*threat* (*F_1, 91_* = 6.865, *p*= 0.010, *η_p_*^2^= 0.070). The ANOVA for threat ratings showed a main effect of *threat* (*F_1, 91_* = 262.186, *p*< 0.001, *η_p_*^2^= 0.742). Therefore, participants rated faces revealing highly intensive threat as more threatening than faces of low-intensity threat. There furthermore was a significant interaction of *threat*sex* (*F_1, 91_* = 8.555, *p*= 0.004, *η_p_*^2^= 0.086). Post-hoc tests showed a significant difference between low and high threat-intensities for women (*p*< 0.001), and for men (*p*< 0.001). For low threat, there was a difference between men and women at trend level (*p*= 0.075), indicating that women gave lower ratings to faces of low threat-intensity than men. There was no effect for high threat between men and women (*p*= 0.362). The ANOVA for threat ratings yielded no significant interaction effect of *attractiveness*threat* (*F_1, 91_* = 1.173, *p*= 0.282, *η_p_*^2^= 0.013).

| Ratings for attractiveness and threat | | high attractiveness &  low threat | low  attractiveness &  low threat | high attractiveness &  high threat | low attractiveness &  high threat |
| --- | --- | --- | --- | --- | --- |
| Women  *N*= 46 | Attractiveness | *M* = 3.71  *SD* = 0.73 | *M* = 2.44  *SD* = 0.75 | *M* = 3.48  *SD* = 0.92 | *M* = 2.44  *SD* = 0.74 |
|  | Threat | *M* = 2.49  *SD* = 0.73 | *M* = 2.46  *SD* = 0.74 | *M* = 4.05  *SD* = 1.05 | *M* = 3.91  *SD* = 1.04 |
| Men  *N*= 47 | Attractiveness | *M* = 4.13  *SD* = 0.67 | *M* = 3.05  *SD* = 0.72 | *M* = 3.75  *SD* = 0.65 | *M* = 2.74  *SD* = 0.67 |
|  | Threat | *M* = 2.63  *SD* = 0.73 | *M* = 2.86  *SD* = 0.83 | *M* = 3.69  *SD* = 1.00 | *M* = 3.89  *SD* = 1.07 |

Table S2: Mean values and standard deviations for attractiveness and threat ratings for the 88 stimuli in the main study. Forty-six women rated male stimuli and 47 men rated female stimuli.

**Results – Sex, Facial Phenotypes, and Trust**

| Trust decisions | high attractiveness  &  low threat | low attractiveness  &  low threat | high attractiveness  &  high threat | low attractiveness  &  high threat |
| --- | --- | --- | --- | --- |
| Women  *N*= 46 | *M* = 0.76  *SD* = 0.21 | *M* = 0.62  *SD* = 0.26 | *M* = 0.48  *SD* = 0.30 | *M* = 0.42  *SD* = 0.30 |
| Men  *N*= 47 | *M* = 0.69  *SD* = 0.27 | *M* = 0.56  *SD* = 0.30 | *M* = 0.53  *SD* = 0.30 | *M* = 0.39  *SD* = 0.32 |

Table S3: Mean values and standard deviations for trust decisions in women and men towards the four different phenotypes.

**Results – Sex, Trust and Response Time**

We performed an ANOVA with *response time* as dependent variable (see Table S4 for response times), and *participants’ sex*, *facial* *attractiveness* and *facial* *threat* as independent variables. We found a significant effect of *participants’ sex* (*F_1, 91_* = 7.920, *p*= 0.006, *η_p_*^2^= 0.080), with men revealing faster response times than women. Note that we observed no other significant interactions of *participants’ sex* either with *facial* *attractiveness* (*F_1, 91_* = 2.018, *p*= 0.159, *η_p_*^2^= 0.022) or *facial* *threat* (*F_1, 91_* = 1.621, *p*= 0.206, *η_p_*^2^= 0.017). There was also no significant interaction between *facial* *attractiveness*facial* *threat*participants’ sex* (*F_1, 91_* = 0.951, *p*= 0.332, *η_p_*^2^= 0.010). Therefore, response times did not vary between women and men contingent on the facial phenotype.

| Response times | high attractiveness  &  low threat | low attractiveness  &  low threat | high attractiveness  &  high threat | low attractiveness  &  high threat |
| --- | --- | --- | --- | --- |
| Women  *N*= 46 | *M* = 1789.62  *SD* = 842.25 | *M* = 2036.09  *SD* = 1103.41 | *M* = 2011.93  *SD* = 1085.71 | *M* = 2008.80  *SD* = 1074.46 |
| Men  *N*= 47 | *M* = 1404.96  *SD* = 633.98 | *M* = 1508.68  *SD* = 852.17 | *M* = 1477.93  *SD* = 757.87 | *M* = 1445.59  *SD* = 746.99 |

Table S4: Mean values and standard deviations for response times of trust decisions in female and male participants; broken down by phenotype.

# **References**

1. Walker, M., Schönborn, S., Greifeneder, R. & Vetter, T. The Basel Face Database: A validated set of photographs reflecting systematic differences in Big Two and Big Five personality dimensions. *PLOS ONE* **13**, e0193190 (2018).
